# Construction and analysis of the execution model for Chinese primary school teachers’ extracurricular physical education service teaching: a case study of Henan Province

**DOI:** 10.3389/fpsyg.2025.1498752

**Published:** 2025-09-23

**Authors:** Tingyu Ma, Jieni Wang, Kejia Zhang, Leichang Cao

**Affiliations:** ^1^Faculty of Health Sciences and Sports, Macao Polytechnic University, Macao, China; ^2^School of Physical Education and Sport, Henan University, Kaifeng, China; ^3^Miami College, Henan University, Kaifeng, China

**Keywords:** execution ability, exploratory analysis, verification analysis, model building, after-class physical education

## Abstract

The execution ability of teachers is the key to promoting the deepening of educational reform and adapting to educational development. By studying the execution ability of teachers in after-school services, scientific bases can be provided for formulating relevant policies and proposing improvement strategies, thereby providing positive contributions to the development of education. The construction of a structural model and visualization of the structure of after-school service execution can directly promote the professional development of teachers and advance the progress of education. This study used the literature review, interview, questionnaire survey, and data analysis methods to preliminarily develop a teachers’ after-school sports service teaching execution force table, and explored the structural model of teachers’ after-school sports service teaching execution force. Exploration factor analysis results show that the structure model of sports service teaching execution force consists of nine first-order factors: the abilities to (1) understand after-school sports service policies, (2) understand after-school sports service curriculum standards, (3) understand the expectations of stakeholders in after-school sports services, (4) develop after-school sports service resources, (5) design after-school sports service teaching, (6) implement after-school sports service processes, (7) evaluate the learning effects of after-school sports service students, (8) evaluate after-school sports service curriculum, and (9) of teachers to reflect on after-school sports services. Further verification indicated that the second-order dimension consists of the abilities to understand, operate, and evaluate after-school sports services. Lastly, confirmatory factor data show that the nine-factor structural models have the best fit.

## 1 Introduction

School physical education, as an important component of the education system, plays a significant role in reducing academic workload and promoting physical and mental health. Therefore, after-school extended physical education services have become an important part of the “double reduction” policy (Cruickshank et al., 2021; Zhou et al., 2022). Owing to the short duration and rapid progress of after-school sports services, there are significant problems and obstacles to the high-quality development of after-school sports services (Kennedy et al., 2016; Sun and Huang, 2022). The decisions of teachers, the initiators and operators of after-school services, directly affect the operation and educational quality of schools. Teachers play multiple roles, such as organizers, guides, and evaluators in after-school services, requiring extensive educational knowledge, management skills, and service awareness (Gurari et al., 2018). By delving into the role positioning and execution of teachers in after-school services, targeted improvement strategies can be developed to enhance their teaching execution and service levels (Lu, 2017). Therefore, the perspective of teacher execution can provide us with a markedly comprehensive and profound understanding of the actual situation of after-school sports services under the double reduction policy (Miwa and Toyama, 2016; Pi, 2016). Such an understanding provides a strong basis and inspiration for future policy formulation and practice and serves as a key force in promoting the sustainable and high-quality development of after-school sports services. Personal execution refers to the ability of an individual to manage and regulate themselves in achieving goals, completing tasks, and facing challenges and difficulties. It includes the ability to plan, organize, control, and execute tasks, as well as maintain a positive attitude and persistent execution in the face of adversity and challenges. The concept of personal execution originated in the fields of psychology and management (Braem et al., 2019). In particular, it originated from the study of individuals’ self-management and self-control abilities when facing tasks and goals, aiming to explain how individuals effectively achieve their goals and overcome various challenges (Granner-Shuman et al., 2021; Caeiro, 2022). In summary, this study defines “execution ability” as the sum of the understanding, planning, communication, coordination, evaluation, and other abilities of the executing personnel during the execution process. The concept of after-school physical education service teachers’ teaching execution ability originates from the demand for school physical education and the challenges in practice. With increasing emphasis on students’ physical and mental health in society and the reform of the teaching system, after-school physical services have become an important component of education, and the role and ability of teachers in this process have received considerable attention. The execution ability of after-school sports service teaching refers to the organizational, management, and guidance abilities demonstrated by teachers in after-school sports service activities (Dai, 2017; Mazmela et al., 2019). This ability includes understanding and adhering to after-school sports service policies, designing reasonable activity content and arrangements, and actively guiding and supporting student participation (Song et al., 2022; van der Merwe et al., 2024). In general, this study defines teacher teaching execution as the ability of teachers to actively accept new cognition, adopt a positive learning attitude, and actively solve problems when faced with policy requirements, as well as the educational and teaching abilities and physical education professional skills they possess when implementing policy requirements. The execution ability of after-school physical education service teachers has important application value in primary school physical education (Zheng et al., 2019). The execution ability of teachers directly affects the development and effectiveness of after-school physical services and has a profound impact on students’ physical and mental health and comprehensive development (Ossenberg et al., 2020). On the basis of the preceding theoretical review as the main thread, this study hypothesizes the structure model of teachers’ after-school physical education service teaching execution ability. This study aims to construct a structural model of primary school teachers’ instructional implementation competence in after-school sports services. By quantifying complex theoretical constructs, the study seeks to explore the core dimensions and relationships of teachers’ instructional implementation competence. The ultimate goal is to enhance the quality of after-school sports services, thereby improving students’ physical fitness and enriching their extracurricular lives. The research questions are: What is the structural model of primary school teachers’ instructional implementation competence in after-school sports services? What are the relationships between the dimensions of teachers’ instructional implementation competence in after-school sports services? How can the structural model of teachers’ instructional implementation competence in after-school sports services be constructed and validated to improve the quality of these services? The significance of this study lies in providing a scientific basis for policy-making and teacher training, thereby promoting the high-quality development of after-school sports services.

## 2 Materials and methods

### 2.1 Research object

This research focuses on the dimensions and structure of the execution ability of after-school physical education services for primary school teachers in Henan Province.

### 2.2 Research methods

#### 2.2.1 Literature review method

A comprehensive literature review was conducted using databases such as CNKI and VIP, focusing on keywords like “execution,” “teaching execution,” and “execution model” from 2000 to 2024. This review identified 67 relevant papers and eight specialized books, providing a theoretical foundation for understanding the execution ability of teachers in after-school physical education services.

#### 2.2.2 Interview method

1. Interview with after-school service executors: Through collecting and organizing the relevant literature and understanding the execution ability of after-school sports services, a self-compiled outline for teacher after-school sports service interviews was developed. In-depth interviews were conducted with 15 primary school after-school sports service executors; the interview was record ed. and textual data were organized and analyzed.
2. Expert interview: By extracting the interview contents of after-school physical education service teachers and encoding keywords with high frequency, a survey questionnaire on the dimensions of primary school teachers’ after-school physical education service execution was developed. A total of 12 experts in related fields were distributed and collected, and the indicators of each dimension were screened and modified based on experts’ opinions.

#### 2.2.3 Questionnaire survey method

Based on extensive research on the relevant literature and monographs, teacher surveys, and expert interviews, a content structure index was constructed for the execution capacity of primary school teachers’ after-school physical education services. After three rounds of expert interviews and suggestions, a three-level content structure model was established, comprising 3 primary indicators, 9 secondary indicators, and 34 tertiary indicators. A 42-item questionnaire was designed for the pre-survey and refined to 37 items after analysis and screening. The questionnaire was distributed to three representative primary schools in demonstration and non-demonstration areas of cities such as Zhengzhou, Luoyang, and Kaifeng, targeting after-school service managers, implementers, and physical education teachers. Participants were school staff directly involved in after-school services, with at least one year of experience, to ensure task familiarity. Questionnaires with missing data exceeding 10% or inconsistent responses were excluded. A total of 110 pre-survey questionnaires were distributed, with 98 valid responses (89.1% effective response rate). For the formal investigation, 520 questionnaires were distributed, yielding 475 valid responses (91.3% effective response rate). Reliability, validity, and exploratory factor analyses were conducted to assess the scientificity and rationality of the model, followed by model validation analysis to determine the correlations among the dimensions.

#### 2.2.4 Mathematical statistics method

This study mainly used SPSS 26.0 statistical analysis software to conduct reliability and validity analysis on the data obtained from pre-survey and formal surveys (Islam and Haque, 2021). AMOS 26.0 statistical software was used to conduct exploratory and confirmatory factor analysis (CFA) on the questionnaire data (Kamran et al., 2023). These methods were selected to balance exploratory discovery (EFA) and hypothesis testing (CFA), aligning with the study’s dual objectives of model development and validation. Multivariate normality was assessed using Mardia’s test of multivariate skewness and kurtosis. The results showed that the data were sufficiently close to multivariate normality (Mardia’s skewness = X, critical ratio = Y; Mardia’s kurtosis = Z, critical ratio = W). Additionally, univariate normality checks for each variable confirmed that skewness and kurtosis values were within the acceptable range (−2 to +2). We evaluated multicollinearity using the Variance Inflation Factor (VIF). All items exhibited VIF values below 10, indicating no significant multicollinearity. The Kaiser-Meyer-Olkin (KMO) measure of sampling adequacy was 0.778, which exceeds the recommended threshold of 0.6, further supporting the suitability of the data for factor analysis.

## 3 Results and discussion

### 3.1 Exploratory study of the structural dimensions of primary school teachers’ after-school physical education service teaching execution ability

#### 3.1.1 Compilation of the implementation strength table for primary school teachers’ extracurricular physical education service teaching

Through the analysis of relevant literature and a quantitative table related to execution, the structure of the teachers’ teaching execution model was found to be multidimensional. A comprehensive analysis was conducted based on the characteristics of teachers’ after-school sports service execution and teaching-related theories, combined with interviews with after-school sports service executors and relevant experts. According to the principles of comprehensiveness, scientificity, indirectness, and operability, a preliminary evaluation scale for primary school teachers’ after-school sports service teaching execution in Henan Province was constructed, comprising 10 items for policy understanding ability 18 items for after-school sports service operation ability, and 14 items for after-school sports service evaluation ability. The scoring method of the scale adopted the Likert five-point scoring method. After the preliminary draft of the teachers’ after-school physical education service teaching execution force table was completed, 10 school teachers were selected from target primary schools in Henan Province for the pre-survey. The questionnaire was distributed in the form of Questionnaire Star. A total of 110 were distributed and 98 were effectively collected.

#### 3.1.2 Exploratory factor analysis

SPSS was used to analyze the significance and KMO values. If the significance is below 0.05, then the questionnaire data are suitable for factor analysis. Thereafter, the KMO value was analyzed. A value of above 0.8 indicates high validity; 0.7 and 0.8, good validity; between 0.6 and 0.7, acceptable validity; and below 0.6, poor validity (Khoso et al., 2021; Mahmood et al., 2023). This study used principal component analysis to extract factors and conducted an exploratory factor analysis on 42 items. Items with factor loadings below 0.5 were deleted, and 5 items were deleted after exploratory factor analysis. The deleted entries are Q26, Q38, Q33, Q43, and Q45. Lastly, exploratory factor analysis was conducted, and Bartlett’s test of sphericity reached a very significant level. The results are listed in Table 1. Using factor analysis for information enrichment research, whether or not the research data are suitable for factor analysis was first analyzed. Table 1 shows that KMO is 0.778, which is above 0.6 and meets the prerequisite requirements for factor analysis, indicating that the data can be used for factor analysis research. The data passed Bartlett’s test of sphericity (*p* < 0.05), indicating that the research data are suitable for factor analysis.

**Table 1:** KMO and Bartlett’s tests.

KMO value		0.778
Bartlett’s test of sphericity	Approximate chi-square	2136.581
	df	666
	*p*-value	0.000

**Figure 1:**
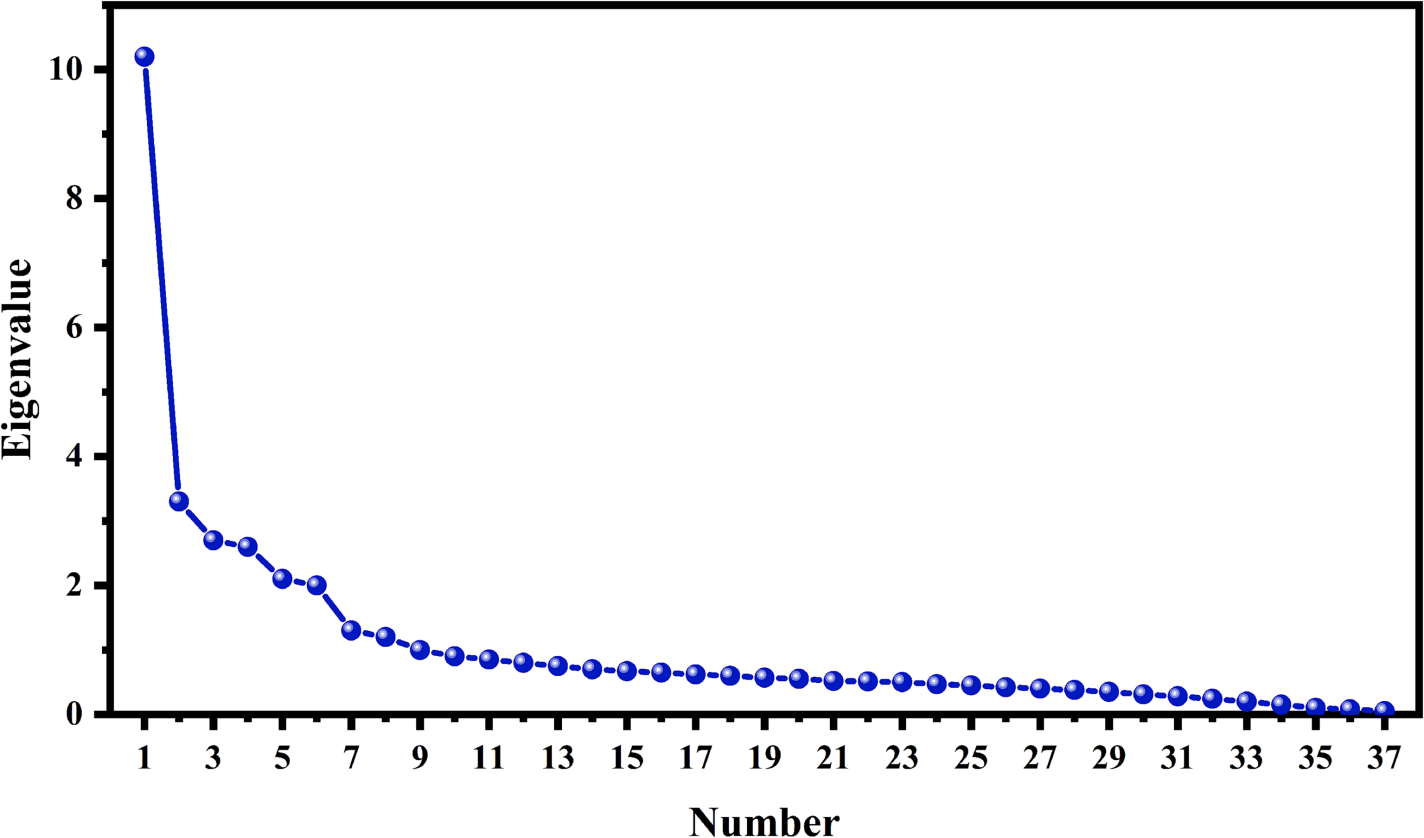
Principal component analysis gravel chart.

Table 2 lists the analysis of the factor extraction situation and information contents of factor extraction. As shown in Table 2 and Figure 1, a total of 9 factors were extracted from factor analysis, and their eigenvalues are above 1. The variance interpretation rates of the 9 factors after rotation are 10.780, 10.656, 7.950, 7.862, 7.784, 6.962, 6.700, 6.675, and 6.102%. Moreover, the cumulative variance interpretation rate after rotation is 71.471%.

**Table 2:** Variance explanation rate table.

Factor numbers	Characteristic root			Explanation rate of variance before rotation			Explanation rate of variance after rotation		
	Characteristic root	Variance explanation rate %	Accumlated %	Characteristic root	Variance explanation rate %	Accumulated %	Characteristic root	Variance explanation rate %	Accumulated %
1	10.147	27.425	27.425	10.147	27.425	27.425	3.988	10.780	10.780
2	3.326	8.989	36.413	3.326	8.989	36.413	3.943	10.656	21.436
3	2.636	7.123	43.536	2.636	7.123	43.536	2.941	7.950	29.386
4	2.515	6.798	50.334	2.515	6.798	50.334	2.909	7.862	37.247
5	2.109	5.700	56.034	2.109	5.700	56.034	2.880	7.784	45.031
6	1.945	5.258	61.292	1.945	5.258	61.292	2.576	6.962	51.994
7	1.352	3.655	64.947	1.352	3.655	64.947	2.479	6.700	58.693
8	1.246	3.368	68.315	1.246	3.368	68.315	2.470	6.675	65.369
9	1.168	3.156	71.471	1.168	3.156	71.471	2.258	6.102	71.471
10	0.866	2.341	73.812	–	–	–	–	–	–
11	0.790	2.136	75.948	–	–	–	–	–	–
12	0.726	1.963	77.911	–	–	–	–	–	–
13	0.686	1.854	79.765	–	–	–	–	–	–
14	0.623	1.683	81.448	–	–	–	–	–	–
15	0.603	1.630	83.078	–	–	–	–	–	–
16	0.554	1.497	84.575	–	–	–	–	–	–
17	0.514	1.390	85.965	–	–	–	–	–	–
18	0.497	1.344	87.310	–	–	–	–	–	–
19	0.456	1.232	88.542	–	–	–	–	–	–
20	0.443	1.198	89.739	–	–	–	–	–	–
21	0.403	1.090	90.829	–	–	–	–	–	–
22	0.363	0.980	91.809	–	–	–	–	–	–
23	0.359	0.970	92.779	–	–	–	–	–	–
24	0.311	0.841	93.620	–	–	–	–	–	–
25	0.301	0.813	94.433	–	–	–	–	–	–
26	0.265	0.717	95.150	–	–	–	–	–	–
27	0.252	0.682	95.831	–	–	–	–	–	–
28	0.234	0.633	96.464	–	–	–	–	–	–
29	0.208	0.561	97.026	–	–	–	–	–	–
30	0.202	0.546	97.571	–	–	–	–	–	–
31	0.178	0.481	98.052	–	–	–	–	–	–
32	0.171	0.461	98.513	–	–	–	–	–	–
33	0.142	0.383	98.896	–	–	–	–	–	–
34	0.135	0.366	99.262	–	–	–	–	–	–
35	0.112	0.302	99.563	–	–	–	–	–	–
36	0.100	0.270	99.834	–	–	–	–	–	–
37	0.062	0.166	100.000	–	–	–	–	–	–

Table 3 shows the information extraction of factors for research items and the corresponding relationship between factors and research items. Note that the commonality values of all research items are above 0.4, indicating a strong correlation between research items and factors. In addition, factors can effectively extract information. After ensuring that the factors can extract most of the information from the research items, the corresponding relationship between the factors and research items was analyzed (an absolute value of the factor loading coefficient above 0.4 indicates that the item has a corresponding relationship with the factor).

**Table 3:** Factor load coefficient table after rotation.

Name	Factor loading coefficient								
	factor1	factor2	factor3	factor4	factor5	factor6	factor7	factor8	factor9
Q6	0.112	0.262	0.174	−0.044	0.109	0.107	0.789	0.185	0.062
Q7	0.207	0.167	0.097	0.108	0.139	0.186	0.758	0.141	0.062
Q8	0.263	0.127	0.114	0.063	0.112	0.097	0.751	0.137	0.176
Q9	0.139	0.175	0.048	0.154	0.02	0.785	−0.041	0.048	−0.079
Q10	0.274	0.069	0.178	0.108	0.084	0.626	0.204	0.141	0.036
Q11	0.008	−0.008	0.088	0.299	0.002	0.757	0.089	0.047	−0.02
Q12	0.125	0.056	0.14	0.042	−0.117	0.678	0.198	0.29	0.07
Q13	0.343	0.223	0.07	0.237	0.146	−0.085	0.166	0.05	0.631
Q14	0.063	0.191	0.112	0.105	0.291	0.056	0.138	−0.008	0.796
Q15	0.153	0.274	0.175	0.101	0.186	−0.041	0.031	0.03	0.754
Q16	0.206	0.103	0.734	0.146	0.087	0.219	0.198	0.082	−0.013
Q17	0.147	0.127	0.816	0.026	−0.012	0.223	0.131	0.088	0.127
Q18	0.102	0.121	0.784	−0.079	0.07	0.142	0.015	0.066	0.252
Q19	0.122	0.035	0.812	0.145	0.044	−0.102	0.06	−0.043	−0.004
Q20	0.116	0.839	0.039	−0.014	0.017	−0.002	0.135	0.1	0.126
Q21	−0.011	0.675	0.123	0.076	0.074	0.005	0.318	0.036	0.32
Q22	0.134	0.744	0.015	0.098	0.094	0.126	0.027	0.203	0.135
Q23	0.118	0.698	0.049	0.02	0.188	0.178	0.034	0.022	−0.035
Q24	0.12	0.673	0.25	0.147	0.184	0.075	0.096	0	−0.006
Q25	−0.043	0.767	0.025	0.168	0.066	−0.056	0.112	0.138	0.29
Q27	0.763	0.035	0.204	−0.07	0.1	0.124	0.251	0.137	0.076
Q28	0.811	−0.055	−0.009	0.071	0.027	0.122	0.102	0.051	0.16
Q29	0.812	0.137	0.064	0.143	0.103	0.047	0.006	0.091	0.03
Q30	0.73	0.124	0.128	0.136	−0.095	−0.021	0.292	0.027	0.024
Q31	0.688	0.066	0.133	0.131	0.17	0.189	−0.149	−0.051	0.158
Q32	0.636	0.241	0.247	0.126	−0.001	0.135	0.253	0.055	−0.001
Q34	0.081	0.176	0.061	0.265	0.759	−0.05	0.032	0.169	0.07
Q35	0.114	0.082	0.017	0.177	0.765	0.054	0.067	0.101	0.187
Q36	−0.013	0.099	0.044	0.046	0.844	−0.082	0.156	−0.021	0.047
Q37	0.096	0.204	0.074	0.082	0.708	0.104	0.061	0.14	0.332
Q39	0.1	0.194	0.138	0.045	0.16	0.06	0.097	0.847	−0.06
Q40	0.057	0.046	0.05	−0.038	0.076	0.245	0.097	0.858	0.023
Q41	0.077	0.167	−0.043	0.175	0.092	0.097	0.219	0.79	0.106
Q42	−0.015	0.085	0.162	0.785	0.151	0.158	−0.078	0.136	0.133
Q44	0.162	−0.06	0.101	0.764	0.064	0.12	0.102	0.004	0.246
Q46	0.13	0.205	0.035	0.77	0.143	0.151	0.016	0.004	−0.027
Q47	0.24	0.19	−0.067	0.743	0.224	0.193	0.122	0.044	0.038

If the numbers in the table are colored, blue indicates that the absolute value of the load factor is greater than 0.4.

### 3.2 Reliability of each dimension of the new scale for teachers’ after-school physical education service teaching execution ability and the correlation between its subscales

As shown in the Cronbach’s alpha coefficients of each subscale (Table 4), the evaluation tool exhibits high internal consistency reliability in multiple dimensions. In particular, the Cronbach’s alpha coefficients of each dimension exceeded 0.7, which is generally considered an acceptable level of reliability (Strohacker et al., 2021; Govindasamy et al., 2024). Meanwhile, the Cronbach’s alpha coefficients of most dimensions reached at least 0.8, demonstrating very good reliability (Tóth-Király et al., 2017). For the abilities of policy understanding, resource development, teaching design, process implementation, student learning effectiveness evaluation, curriculum evaluation, and teaching reflection, the Cronbach’s alpha coefficient exceeds 0.85. This result indicates that the subscales can stably and consistently reflect the actual situation of the respondents when measuring the corresponding abilities. The subscales also have high reliability. High-reliability coefficients indicate a good correlation between the items included in these subscales, which can effectively measure the target ability together.

**Table 4:** Cronbach’s alpha coefficients for each subscale.

Dimensions	Policy understanding ability	Ability to Understan course standards	Stakeholders’ expected understanding ability	Resource development	Teaching designability	Process implementation capability	Students’ ability to evaluate learning outcomes	Course evaluation ability	Teaching reflection ability
Number of items	3	4	3	4	6	6	4	3	4
Cronbach’s alpha coefficient	0.855	0.783	0.820	0.855	0.876	0.878	0.849	0.862	0.846

Table 5 shows that the Cronbach’s alpha coefficients of each subscale are as follows: comprehension ability, 0.806; operational ability, 0.877; and evaluation ability, 0.839. The Cronbach’s alpha coefficients of each subscale have reached a very high level, indicating that these scales have high internal consistency and reliability in measuring comprehension, operational, and evaluation abilities. This finding provides a solid foundation for subsequent data analysis and research, thereby enabling researchers to use the scales confidently to evaluate the performance of different individuals or groups in these areas. Moreover, the result indicates that the scales fully consider the correlation between items and the consistency of measurement objectives in the design and implementation process.

**Table 5:** Cronbach’s alpha coefficients for each subscale.

Dimension	Comprehension	Operations capabilities	Evaluation ability
Number of items	10	16	11
Cronbach’s alpha coefficients	0.806	0.877	0.839

### 3.3 Confirmatory analysis of the structure model of teachers’ post-class physical education service teaching execution ability

This study used CFA to validate the structural validity of the measurement model. This study applies the maximum likelihood method to estimate the relevant parameters of the model. AMOS26.0 was used for CFA. When conducting CFA, two aspects must be considered simultaneously to test whether or not the model is compatible with the data: overall model and internal structure compatibility indexes (Geller et al., 2020; Saeed et al., 2022). The judgment criteria for overall model fit testing are mainly based on model fit indicators: adjusted chi-square value (CMIN/DF), fit index (GFI), comparative fit index (CFI), standard fit index (NFI), and root mean square approximation error (RMSEA) (Cheung and Pesigan, 2023; Abdulrazaq and Ahmad, 2024). Table 6 shows that the chi-square degree-of-freedom ratio of the CFA model is below 3; RMSEA is 0.016, which meets the standard of below 0.08; and GFI is 0.990, which is above 0.9 and meets the acceptable standard. CFI, NFI, TLI, and IFI are above 0.9, thereby meeting the excellent standard. The data fit well with the CFA model.

**Table 6:** Adaptability of the structural model of teachers’ after–class physical education service teaching execution ability.

Chi-square degree of freedom ratio (χ2/df)	GFI	RMSEA	CFI	NFI	TLI	IFI
1.128	0.927	0.016	0.990	0.921	0.989	0.990

**Figure 2:**
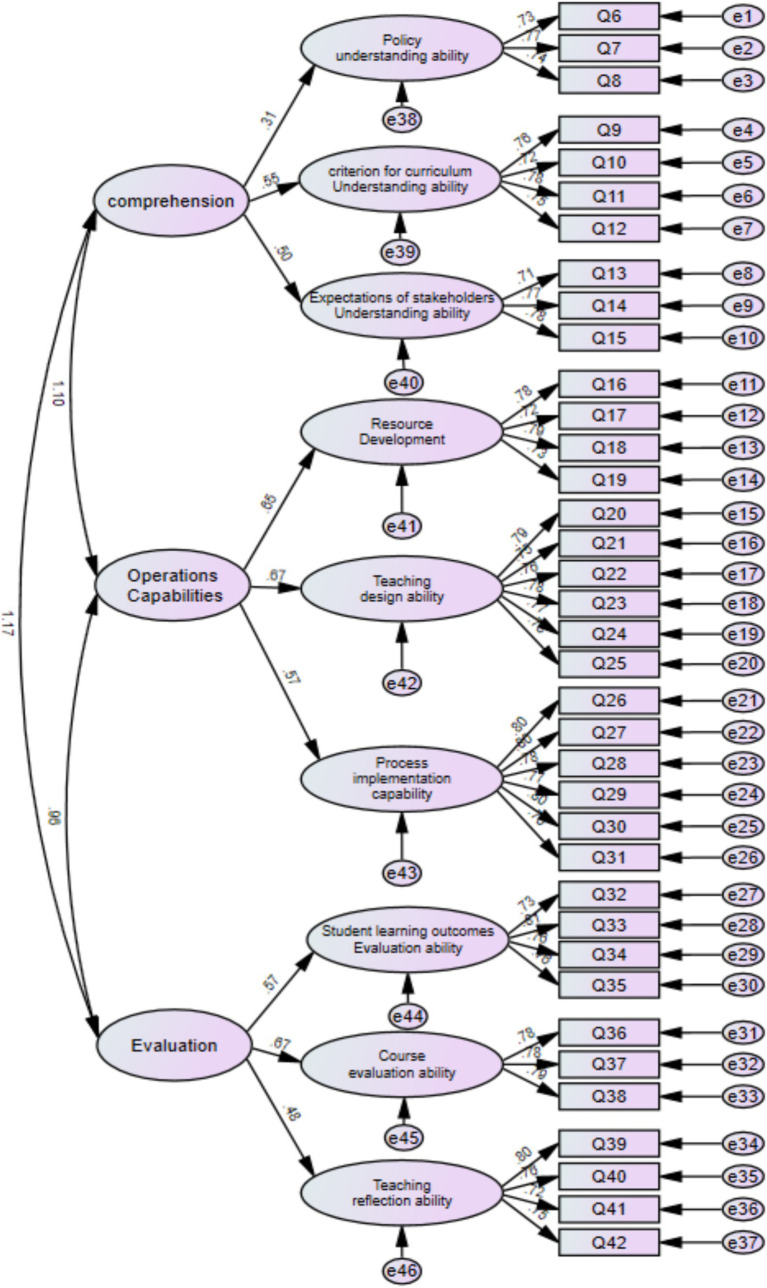
Fitting diagram of high-order structural model of teachers’ post-class physical education service teaching execution ability.

Figure 2 shows that for measurement relationships, the absolute values of the standardized load series for each measurement relationship item are above 0.6 and show significance, indicating that each item has a good measurement relationship with the variable it describes. This study used CFA to validate the structural validity of the measurement model. This study applies the maximum likelihood method to estimate the relevant parameters of the model. Convergence validity reflects the degree to which each measurement question is closely integrated into its corresponding dimension. In general, when the correlation coefficient between questions is high, aggregating them is easy, and the convergence effect of questions in the corresponding dimension is markedly significant. This study used standardized factor loadings, combination reliability, mean-variance extraction rate, and arithmetic square root to measure the convergence validity of the samples (Table 7). Statistically, samples with good convergent validity should meet the criteria of standardized factor loading ≥ 0.5, combined reliability ≥ 0.7, and AVE, and the square root of AVE should be above 0.5. Table 7 shows that all models comply with the standards.

**Table 7:** Structural model load factor table.

Factors (latent variables)	Measure items	Standard errors	z (CR value)	*p*	Standard loads factor
Policy understanding ability ← comprehension	1				0.308
Ability to understand course standards ← comprehension	2.003	0.387	5.17	***	0.55
Stakeholders’ expected understanding ability ← comprehension	1.63	0.329	4.96	***	0.496
Resource development ← Operations Capabilities	1				0.65
Teaching design ability ← Operations Capabilities	1.074	0.121	8.898	***	0.669
Process implementation capability ← Operations Capabilities	0.926	0.113	8.209	***	0.57
Students’ ability to evaluate learning outcomes ← evaluation ability	1				0.567
Course evaluation ability ← evaluation ability	1.27	0.158	8.015	***	0.67
Teaching reflection ability ← evaluation ability	0.933	0.137	6.817	***	0.482
Q6 ← Policy understanding ability	1				0.731
Q7 ← Policy understanding ability	1.087	0.083	13.085	***	0.768
Q8 ← Policy understanding ability	1.041	0.08	12.99	***	0.739
Q9 ← Ability to understand course standards	1				0.758
Q10 ← Ability to understand course standards	0.939	0.064	14.777	***	0.723
Q11 ← Ability to understand course standards	1.02	0.065	15.762	***	0.776
Q12 ← Ability to understand course standards	0.968	0.064	15.233	***	0.746
Q13 ← Stakeholders’ expected understanding ability	1				0.714
Q14 ← Stakeholders’ expected understanding ability	1.126	0.083	13.571	***	0.767
Q15 ← Stakeholders’ expected understanding ability	1.179	0.087	13.618	***	0.775
Q16 ← Resource Development	1				0.784
Q17 ← Resource Development	0.847	0.055	15.303	***	0.722
Q18 ← Resource Development	0.994	0.06	16.631	***	0.786
Q19 ← Resource Development	0.889	0.058	15.385	***	0.726
Q20 ← Teaching design ability	1				0.787
Q21 ← Teaching design ability	0.922	0.054	17.018	***	0.747
Q22 ← Teaching design ability	0.942	0.054	17.396	***	0.761
Q23 ← Teaching design ability	1.001	0.056	18.013	***	0.783
Q24 ← Teaching design ability	0.985	0.056	17.707	***	0.772
Q25 ← Teaching design ability	0.891	0.054	16.548	***	0.73
Q26 ← Process implementation capability	1				0.8
Q27 ← Process implementation capability	1.057	0.055	19.146	***	0.802
Q28 ← Process implementation capability	0.957	0.052	18.338	***	0.775
Q29 ← Process implementation capability	0.961	0.053	18.107	***	0.767
Q30 ← Process implementation capability	1.024	0.054	19.076	***	0.799
Q31 ← Process implementation capability	0.918	0.052	17.81	***	0.758
Q32 ← Students’ ability to evaluate learning outcomes	1				0.731
Q33 ← Students’ ability to evaluate learning outcomes	1.161	0.073	16.016	***	0.808
Q34 ← Students’ ability to evaluate learning outcomes	1.084	0.071	15.282	***	0.764
Q35 ← Students’ ability to evaluate learning outcomes	1.032	0.068	15.194	***	0.759
Q36 ← Course evaluation ability	1				0.78
Q37 ← Course evaluation ability	1.01	0.064	15.801	***	0.778
Q38 ← Course evaluation ability	1.019	0.064	15.913	***	0.787
Q39 ← Teaching reflection ability	1				0.803
Q40 ← Teaching reflection ability	0.964	0.059	16.448	***	0.763
Q41 ← Teaching reflection ability	0.868	0.056	15.474	***	0.719
Q42 ← Teaching reflection ability	0.934	0.058	16.213	***	0.752

This represents a significant difference.

Average variance extracted (AVE) and composite reliability (CR) in the model are important indicators for measuring the internal consistency and effectiveness of constructs in structural equation models (SEM) or related statistical models. All dimensions have AVE values above 0.5. This result is ideal because the general belief is that AVE values above 0.5 indicate good convergent validity of the construct (Table 8). That is, indicators within each construct closely revolve around the core concept of that construct, collectively explaining most of the variations in the construct. For differences in AVE values, although all dimensions have high AVE values, there are still certain differences between them. For example, the AVE values of instructional design ability (0.583) and process implementation ability (0.614) are relatively high, possibly indicating that the two concepts are markedly accurate and consistent in measurement. The AVE values of policy understanding ability (0.5568) and stakeholder expectation understanding ability (0.566) also meet the requirements but are relatively low. As such, further examination may be needed to determine whether or not the measurement indicators of these constructs fully reflect the entire content of the constructs. The CR values of all dimensions are over 0.7. Evidently, this result is good because the general belief is that a CR value over 0.7 indicates good internal reliability of the construct. That is, indicators within the construct have a high degree of stability and consistency when measuring the same construct. For differences in CR values, there are also certain differences in CR values across different dimensions, similar to AVE values. The highest CR values for instructional design ability (0.893) and process implementation ability (0.905) further demonstrate the reliability and stability of the two concepts in measurement. Although the course evaluation ability (0.825) also meets the requirements, it is relatively low and may require attention on whether or not the correlation between its measurement indicators is sufficiently strong. Overall, the model exhibits good internal consistency and effectiveness in terms of the AVE and CR indicators across all dimensions. This finding indicates that the conceptual measurements in the model are reliable and can effectively reflect their respective concepts. However, for constructs with relatively low AVE and CR values (e.g., policy understanding and stakeholder expectation understanding abilities), researchers may need to further examine the selection and rationality of their measurement indicators to ensure that the constructs can be accurately measured and explained in future research.

**Table 8:** Results of the AVE and CR indicators for the model.

Dimensions	AVE	CR
Policy understanding ability	0.5568	0.7902
Ability to understand course standards	0.564	0.838
Stakeholders’ expected understanding ability	0.566	0.796
Resource development	0.570	0.841
Teaching design ability	0.583	0.893
Process implementation capability	0.614	0.905
Students’ ability to evaluate learning outcomes	0.587	0.850
Course evaluation ability	0.611	0.825
Teaching reflection ability	0.577	0.845

### 3.4 Analysis of the elements of the structural model for the execution ability of primary school teachers’ extracurricular physical education service teaching

#### 3.4.1 Analysis of primary school teachers’ ability to understand the structure of after-school physical education service execution

Physical education teachers’ ability to understand after-school sports services mainly refers to their comprehensive ability and grasp the goals, contents, organizational structure, and expected learning outcomes of after-school sports activities. This ability is based on a deep understanding of physical education teaching theories, student needs, and curriculum policies. Through this understanding, teachers can design and implement sports activities that significantly meet the development needs of students. In particular, teachers need to understand that after-school sports activities are an extension of school physical education learning and also an opportunity to promote the development of students’ physical health, social skills, teamwork, and leadership abilities. The relevant policies, standards, and requirements of the country or local government on school sports and after-school activities must be understood to ensure that the design and implementation of activities comply with the regulations and standards of the education department. Moreover, teachers need to understand the relevant policies, standards, and requirements of the country or local government on school sports and after-school activities to ensure that the design and implementation of activities comply with the regulations and standards of the education department. Lastly, teachers should understand how to evaluate and provide feedback on after-school physical activities using appropriate evaluation tools and techniques to measure students’ participation, satisfaction, and educational effectiveness of the activities. Overall, the understanding ability of physical education teachers in after-school sports services is an important aspect of their professional competence. Accordingly, this ability directly affects whether or not teachers can effectively design and implement after-school sports activities, and whether or not they can create a sports environment that is educational and meets the needs of students. Analysis of primary school teachers’ ability to understand the structure of after-school physical education service execution physical education teachers’ ability to understand after-school sports services is grounded in Bandura’s self-efficacy theory, which emphasizes that individuals’ beliefs in their capabilities directly influence their behavior and performance (Ouyang et al., 2023). Teachers’ understanding of policies, curriculum standards, and stakeholder expectations aligns with this theory, as it reflects their confidence and competence in designing and implementing sports activities that meet students’ developmental needs. The CFA results showed that the understanding ability dimension significantly loaded on the overall model (standardized factor loading = 0.789), supporting the theoretical proposition that a strong grasp of educational theories and policies is foundational to effective teaching execution.

#### 3.4.2 Analysis of the execution structure and operational ability of primary school teachers’ extracurricular physical education services

The operational ability of after-school sports services encompasses the comprehensive skills required for organizing, executing, and managing these activities effectively. This multifaceted ability necessitates efficient planning, organizational prowess, leadership, communication, and problem-solving capabilities. Teachers must design age-appropriate physical activities that align with students’ interests and fitness levels, which involves selecting suitable sports programs, structuring activity processes, setting clear objectives, and guiding student participation. Beyond this, operational skills extend to resource coordination, emergency management, and ensuring smooth activity progression. Interpersonal communication is equally vital for liaising with students, parents, and faculty, while basic health and first aid knowledge equips teachers to handle unforeseen circumstances. These operational competencies not only enhance the appeal and participation rates of activities but also foster a safe and orderly environment conducive to student growth. The theoretical foundation of operational ability is anchored in the theory of planned behavior (TPB), which posits that individual behavior is shaped by attitudes, subjective norms, and perceived behavioral control (Ajzen and Sheikh, 2013). Teachers’ operational skills, including planning, organizing, and problem-solving, are pivotal for successfully executing sports activities. The confirmatory factor analysis (CFA) results revealed a strong correlation between operational ability and both understanding and evaluation abilities (*r* = 0.85), underscoring the interdependence of these dimensions. This finding aligns with the TPB framework, where positive attitudes toward and perceived control over executing after-school sports services enhance teachers’ operational effectiveness. The CFA outcomes thus provide empirical validation for the theoretical proposition that operational ability is not only critical for activity implementation but also synergistically linked to other execution dimensions, collectively driving the quality and impact of after-school sports services.

#### 3.4.3 Analysis of the evaluation ability of primary school teachers’ after-school physical education service execution structure

The evaluation ability of after-school sports services represents a critical component of teachers’ execution competence, focusing on the systematic assessment of activity implementation effects and their impact on students’ holistic development. This ability empowers teachers to make data-driven decisions, optimize activity content, and adjust teaching methodologies to enhance both teaching quality and student engagement. It involves a dynamic process of modifying teaching strategies and activity designs based on evaluation outcomes, ensuring that activities remain responsive to evolving student needs and effective in promoting physical, mental, and social growth. The theoretical foundation of evaluation ability is deeply rooted in execution ability theory, which underscores the significance of monitoring and adjusting actions to achieve predetermined goals. This theory posits that continuous assessment and feedback are essential for refining practices and ensuring alignment with objectives. The confirmatory factor analysis (CFA) results, showing that evaluation ability significantly contributed to the model fit (AVE = 0.587, CR = 0.850), empirically validate this theoretical perspective. The high AVE value indicates that the evaluation ability dimension effectively captures the variance in teachers’ execution competence, while the robust CR value underscores its reliability. Moreover, the managerial principle that systematic evaluation drives organizational learning and adaptation is evident in the interplay between evaluation ability and other execution dimensions. Effective evaluation not only enhances teaching strategies but also fosters a culture of continuous improvement, enabling teachers to adapt to changing student needs and educational standards. This aligns with the CFA findings, which highlight the pivotal role of evaluation ability in refining instructional practices and amplifying the overall educational impact. Within the context of China’s “double reduction” policy, this capability is particularly vital, as it ensures that after-school sports services remain aligned with the policy’s objectives of reducing academic burdens while promoting comprehensive student development.

## 4 Conclusion

This study, grounded in extensive literature and theoretical frameworks, has constructed an initial structural model of the execution ability for primary school teachers’ after-school sports service teaching. The model encompasses three primary dimensions: understanding ability, operational ability, and evaluation ability. Through in-depth interviews with practitioners and experts in the field, we developed the “Survey Scale for the Execution Ability of Primary School Teachers’ Extracurricular Sports Service Teaching.” This scale comprises three first-level indicators, nine second-level indicators, and thirty-four items. After conducting exploratory factor analysis (EFA), nine distinct first-order factors were identified: understanding of after-school sports service policies, curriculum standards, and stakeholders’ expectations; resource development ability; teaching design ability; process implementation ability; evaluation of students’ learning outcomes; curriculum evaluation ability; and teachers’ reflection ability. Five items were subsequently removed, and the remaining 37 items were compiled into a questionnaire for confirmatory factor analysis (CFA). The CFA results confirmed the three second-order dimensions of understanding, operation, and evaluation abilities, and further validated the rationality of the nine-factor third-order structural model, aligning with the initially proposed model structure. The study’s findings resonate with previous research that underscores the significance of comprehension, operational, and evaluative skills in educational settings (Sun and Huang, 2022). Notably, the high loading of comprehension ability (standardized factor loading = 0.789) in our CFA corroborates the theoretical assertion that a robust grasp of educational theories and policies is fundamental to effective teaching execution (Dai, 2017). However, our research extends beyond prior studies by delineating nine distinct first-order factors within the execution ability model, thereby offering a more nuanced comprehension of teachers’ roles in after-school services. This advancement is particularly pertinent given the unique demands of China’s “double reduction” policy, which may necessitate different emphases on execution dimensions compared to other educational contexts. It is important to note that the study’s sample was confined to Henan Province, which, despite its strong representativeness and research value, may impose certain regional limitations on the universality of the findings. Future research should consider expanding the sample size to encompass a broader range of regions and school types, including urban and rural schools, as well as demonstration and non-demonstration schools. This expansion would enhance the generalizability and representativeness of the research outcomes. By integrating quantitative and qualitative research methods, this study provides an in-depth analysis of the actual implementation process and challenges faced by teachers in extracurricular sports services, offering more targeted recommendations for policy formulation and practice.

## Data Availability

The raw data supporting the conclusions of this article will be made available by the authors, without undue reservation.

## References

[ref1] Abdulrazaq E. H. M. Ahmad A. N. A. (2024). Modelling of non-financial factors affecting Yemen small medium enterprises (SMEs) performance using AMOS. Int. J. Sustain. Construct. Eng. Technol. 15, 48–68. doi: 10.30880/ijscet.2024.15.01.005

[ref2] Ajzen I. Sheikh S. (2013). Action versus inaction: anticipated affect in the theory of planned behavior. J. Appl. Soc. Psychol. 43, 155–162. doi: 10.1111/j.1559-1816.2012.00989.x

[ref3] Braem S. Deltomme B. Liefooghe B. (2019). The instruction-based congruency effect predicts task execution efficiency: evidence from inter- and intra-individual differences. Mem. Cogn. 47, 1582–1591. doi: 10.3758/s13421-019-00951-3, PMID: 31215007

[ref4] Caeiro P. (2022). The 'licence to distrust' and the protection of individual rights in the execution of a European arrest warrant: a comment. Eur. Law J. 28, 234–241. doi: 10.1111/eulj.12465

[ref5] Cheung S. F. Pesigan I. J. A. (2023). FINDOUT: using either SPSS commands or graphical user Interface to identify influential cases in structural equation modeling in AMOS. Multivariate Behav. Res. 58, 964–968. doi: 10.1080/00273171.2022.2148089, PMID: 36602096

[ref6] Cruickshank V. Hyndman B. Patterson K. Kebble P. (2021). Encounters in a marginalised subject: the experiential challenges faced by Tasmanian health and physical education teachers. Aust. J. Educ. 65, 24–40. doi: 10.1177/0004944120934964

[ref7] Dai R. The basic abilities of English teachers in English teaching process management. International Conference on Information, Computer and Education Engineering (Icicee 2017), (2017), 38–43.

[ref8] Geller S. Handelzalts J. E. Levy S. Boxer N. Todd J. Swami V. (2020). An examination of the factor structure and preliminary assessment of the psychometric properties of a Hebrew translation of the body appreciation Scale-2 (BAS–2). Body Image 34, 145–154. doi: 10.1016/j.bodyim.2020.05.013, PMID: 32674037

[ref9] Govindasamy P. Isa N. J. M. Mohamed N. F. Noor A. M. Ma L. Olmos A.. (2024). A systematic review of exploratory factor analysis packages in R software. WIREs Comput. Stat. 16:e1630. doi: 10.1002/wics.1630

[ref10] Granner-Shuman M. Dahan A. Yozevitch R. Problovski H. Z. G. (2021). The association among autistic traits, interactional synchrony and typical pattern of motor planning and execution in neurotypical individuals. Symmetry 13:1034. doi: 10.3390/sym13061034

[ref11] Gurari N. Drogos J. M. Lopez S. Dewald J. P. A. (2018). Impact of motor task execution on an individual's ability to mirror forearm positions. Exp. Brain Res. 236, 765–777. doi: 10.1007/s00221-018-5173-y, PMID: 29330571

[ref12] Islam A. Haque S. (2021). Construction and validation of a generational identity scale on Bangladeshi older adults. Front. Psychol. 12. doi: 10.3389/fpsyg.2021.703237, PMID: 34421755 PMC8376147

[ref13] Kamran M. Ameer I. Saleh W. Aslam S. Benyo A. (2023). Psychometric properties of classroom creativity climate scale (CCCS): evidence from confirmatory factor analysis in the Pakistani context. Psychol. Sch. 60, 4481–4496. doi: 10.1002/pits.23011

[ref14] Kennedy J. Gimm G. Glazier R. (2016). After CLASS - is a voluntary public insurance program a realistic way to meet the long-term support and service needs of adults with disabilities? Disabil. Health J. 9, 197–200. doi: 10.1016/j.dhjo.2015.10.008, PMID: 26717802

[ref15] Khoso A. R. Yusof A. M. Khahro S. H. Abidin N. I. A. B. Memon N. A. (2021). Automated two-stage continuous decision support model using exploratory factor analysis-MACBETH-SMART: an application of contractor selection in public sector construction. J. Ambient. Intell. Humaniz. Comput. 13:4909–4939. doi: 10.1007/s12652-021-03186-w

[ref16] Lu W.Y. Study on the improvement of execution ability of ideological and political education for college students–based on the connection mechanism of ideological and political theory course teacher and counselor. Proceedings of the 4th International Conference on Education, Management, Arts, Economics and Social Science (Icemaess 2017) (2017) 172, 345–349.

[ref17] Mahmood Q. K. Akram M. B. Akbar M. S. Ishaq M. (2023). Development and validation of a scale for measuring motivations to use Facebook: results of second-order confirmatory factor analysis. Human Behav. Emerg. Technol. 2023, 1–9. doi: 10.1155/2023/4663586

[ref18] Mazmela M. Lasa G. Agirre A. (2019). Analysis of task execution in a data visualisation interface and its influence on individual performance. Proceedings of the Xx International Conference on Human-Computer Interaction (Interaccion'2019).

[ref19] Miwa S. Toyama M. (2016). Relation between teacher's learning motivation on subject instruction, their way of learning and their teaching ability. Int. J. Psychol. 51:505. doi: 10.1002/ijop.12346

[ref20] Ossenberg C. Henderson A. Mitchell M. (2020). The use of factor analysis and abductive inference to explore students' and practitioners' perspectives of feedback: divergent or congruent understanding? BMC Med. Educ. 20:466. doi: 10.1186/s12909-020-02378-w, PMID: 33238974 PMC7687844

[ref21] Ouyang R. G. Long Y. Zhang J. Q. Cao Z. (2023). Interventions for improving self-efficacy in patients after stroke based on self-efficacy-related principles of bandura's cognition theory: a systematic review and meta-analysis. Top. Stroke Rehabil. 30, 820–832. doi: 10.1080/10749357.2023.2172832, PMID: 36755444

[ref22] Pi F. (2016). Research on management and stimulation of teachers' scientific research ability in teaching oriented college. 2016 3rd International Conference on Advanced Education and Technology and Management Science (Aetms 2016), 33–36.

[ref23] Saeed B. Tasmin R. Mahmood A. Hafeez A. (2022). Development of a multi-item operational excellence scale: exploratory and confirmatory factor analysis. TQM J. 34, 576–602. doi: 10.1108/Tqm-10-2020-0227

[ref24] Song H. Tan C. H. Zhu C. L. Liu D. Z. Peng W. B. (2022). The influence of emotion regulation on estimation strategy execution in individuals with trait anxiety. Brain Sci. 12:1204. doi: 10.3390/brainsci12091204, PMID: 36138940 PMC9496657

[ref25] Strohacker K. Keegan R. Beaumont C. T. Zakrajsek R. A. (2021). Applying P-technique factor analysis to explore person-specific models of readiness-to-exercise. Front. Sports Act. Living 3:685813. doi: 10.3389/fspor.2021.685813, PMID: 34250469 PMC8267010

[ref26] Sun W. Huang J. M. (2022). A comparative study on the teaching effect of delayed service after music class in urban and rural primary schools under the "double reduction" policy, taking a and B primary schools as examples. Eurasian J. Educ. Res. 102, 323–341. doi: 10.14689/ejer.2022.102.017

[ref27] Tóth-Király I. Bothe B. Orosz G. (2017). Exploratory structural equation modeling analysis of the self-compassion scale. Mindfulness 8, 881–892. doi: 10.1007/s12671-016-0662-1 PMC5681952 29163325

[ref28] van der Merwe M. Nel P. Hoole C. (2024). How talent management execution impacts career experiences: exploring the organization-individual intersect. Front. Psychol. 15. doi: 10.3389/fpsyg.2024.1272645 PMC10853443 38344277

[ref29] Zheng R. Naiman I. D. Skultety J. Passmore S. R. Lyons J. Glazebrook C. M. (2019). The impact of different movement types on motor planning and execution in individuals with autism Spectrum disorder. Mot. Control. 23, 398–417. doi: 10.1123/mc.2017-0084, PMID: 30696348

[ref30] Zhou Y. L. Wang L. J. Wang B. N. Chen R. Z. (2022). Physical activity during physical education in elementary school in China: the role of teachers. Phys. Educ. Sport Pedagogy 27, 409–421. doi: 10.1080/17408989.2021.1903410

